# Early vascular changes after silicone oil removal using optical coherence tomography angiography

**DOI:** 10.1186/s12886-023-02868-7

**Published:** 2023-03-29

**Authors:** Yanan Hou, Lei Liu, Gang Wang, Junwei Xie, Yi Wang

**Affiliations:** 1 Department of Fundus, Hefei aier eye Hospital, Hefei, China; 2 Department of Fundus, Chongqing aier eye Hospital, 2 Huatang Road, Huaxin Street, Jiangbei District, 404000 Chongqing, Chongqing, China

**Keywords:** Silicone oil tamponade, Optical coherence tomography, Angiography, Silicone oil removal, Vessel density, Perfusion density

## Abstract

**Background:**

This study evaluated the vascular changes in the macular and peripapillary regions before and after silicone oil (SO) removal in patients with rhegmatogenous retinal detachment.

**Methods:**

This single-center case series assessed patients who underwent SO removal at one hospital. Patients who underwent pars plana vitrectomy and perfluoropropane gas tamponade (PPV + C_3_F_8_) were selected as controls. Superficial vessel density (SVD) and superficial perfusion density (SPD) in the macular and peripapillary regions were assessed by optical coherence tomography angiography (OCTA). Best-corrected visual acuity (BCVA) was assessed using LogMAR.

**Results:**

Fifty eyes were administered SO tamponade, 54 SO tamponade(SOT) contralateral eyes, 29 PPV + C_3_F_8_ eyes, and 27 PPV + C_3_F_8_ contralateral eyes were selected. SVD and SPD in the macular region were lower in eyes administered SO tamponade compared with SOT contralateral eyes (*P* < 0.01). Except for the central area, SVD and SPD in the other areas of the peripapillary region were reduced after SO tamponade without SO removal (*P* < 0.01). No significant differences were found in SVD and SPD between PPV + C_3_F_8_ contralateral and PPV + C_3_F_8_ eyes. After SO removal, macular SVD and SPD showed significant improvements compared with preoperative values, but no improvements in SVD and SPD were observed in the peripapillary region. BCVA (LogMAR) decreased post-operation and was negatively correlated with macular SVD and SPD.

**Conclusions:**

SVD and SPD are decreased during SO tamponade and increased in the macular region of eyes that underwent SO removal, suggesting a possible mechanism for reduced visual acuity during or after SO tamponade.

**Trial registration:**

Registration date: 22/05/2019; Registration number, ChiCTR1900023322; Registration site, Chinese Clinical Trial Registry (ChiCTR).

**Supplementary Information:**

The online version contains supplementary material available at 10.1186/s12886-023-02868-7.

## Introduction

The combination of vitrectomy and silicone oil (SO) tamponade is widely used for treating complex retinal detachment accompanied by proliferative vitreoretinopathy, large retinal tears, and ocular trauma [1–3]. Nevertheless, the long-term use of SO tamponade leads to multiple complications, including silicone emulsification, optic nerve atrophy, poor visual outcome, and ocular hypertension [4–7]. It is recommended that SO tamponade be temporary and removed as soon as possible [8], but there is no consensus on the optimal duration [9]. SO removal is used to remove the oil when judged no longer necessary or to manage the complications of SO tamponade.

Changes in macular and peripapillary microcirculation can affect vision through impaired oxygen metabolism in the retina [10]. The narrowing of arterioles caused by SO might interfere with oxygen delivery in the retina, leading to hypoxia and possibly ischemia [11, 12]. Previous studies mainly focused on optical coherence tomography (OCT) and microperimetry [13, 14]. Reports assessing changes in the retina’s microvascular structures after SO tamponade or SO removal are scarce.

OCT angiography (OCTA) represents a new, noninvasive visualization technique that provides vascular information on macular and peripapillary microcirculation with good repeatability and reproducibility [15]. With this method, retinal and choroidal microvessels can be observed layer by layer. In addition, the retinal microvasculature can be assessed quantitatively at the superficial (SCP) and deep (DCP) capillary plexuses [16]. Two reports published in 2020 had conflicting views on whether SO tamponade duration affects retina vascular density (VD) [17, 18].

Therefore, the present study aimed to evaluate the changes in early superficial vessel density (SVD) and superficial perfusion density (SPD) in the macular and peripapillary regions by OCTA before and after SO removal in patients with macula-off rhegmatogenous retinal detachment (RRD).

## Materials and methods

### Study design and patients

This single-center case series prospectively included consecutive patients who underwent SO removal from June to December 2019 at Chongqing Aier Eye Hospital. Patients who underwent SO removal combined with phacoemulsification and intraocular lens implantation were included. The inclusion criteria were (1) intravitreal SO tamponade in one eye for primary macula-off rhegmatogenous retinal detachment and normal macular morphology of the attached retina, (2) PVR grade A or B, (3) macular involvement, (4) hole size < 2 PD, (5) best-corrected visual acuity (BCVA) in the eye administered SO tamponade > 0.1 using the international standard visual acuity chart, (6) no SO emulsification, (7) clear media in both eyes, (8) without hypotony, (9) without viral retinitis, and (10) without chronic uveitis. The exclusion criteria were: (1) keratopathy, severe cataract, or diabetic retinopathy, (2) macular edema or hole, (3) giant retinal tear, (4) high myopia or retinal atrophy caused by high myopia, (5) glaucoma, (6) secondary epiretinal membrane (ERM), (7) retinal redetachment during follow-up, (8) optical media opacity overtly interfering with OCT imaging, or (9) pediatric retinal detachment. Patients with suspected drug toxic side effects were not excluded because the causality could not be determined with certainty. Eyes with SO tamponade that met the above criteria were selected as the SO tamponade eye group. Contralateral eyes with BCVA (LogMAR) < 0.1 and OCTA signal strength above 8 were selected as control eyes.

When the current study was first designed, there was no perfluoropropane (C_3_F_8_) supply in China, which became available in January 2020, when our hospital began using C_3_F_8_. Thus, the patients who underwent pars plana vitrectomy (PPV) and C_3_F_8_ gas tamponade from February to June 2020 at Chongqing Aier Eye Hospital were also selected as controls. In order to avoid bias, the same eligibility criteria described above for patients with SO tamponade were applied to patients with C_3_F_8_.

The study was performed according to the Declaration of Helsinki. It was approved by the Ethics Committee of Chongqing Aier Eye Hospital (EC approval number: IRB2019009). All participants provided signed informed consent. Clinical trial registration: Registration date: May 22, 2019; Registration number, ChiCTR1900023322; Registration site, Chinese Clinical Trial Registry (ChiCTR).

### Surgical procedures

SO removal was performed under retrobulbar anesthesia by associate chief physicians (L.L. and G.W.) using the 23-G Constellation device (Alcon, Fort Worth, TX, USA) and trocars in the inferotemporal and superior quadrants 3.5 mm from the limbus. Infusion liquid was used for oil exchange. An infusion line was inserted into the inferotemporal port, and all the SO was removed using a supratemporal approach with the vacuum set at 450–600 mmHg. Phacoemulsification and intraocular lens implantation through a 2.75-mm micro-coaxial incision were performed when needed (n = 3 patients in the SO group). SO removal was carried out by the pars plana approach upon confirmation of retinal attachment. Following silicone oil removal, the trocars were removed, and the sclerotomies were closed with an 8 − 0 vicryl suture. After SO removal, the patients did not need to follow any position.

Among the 50 patients treated with high-density SO (5000 centistokes, Carl-Zeiss, Germany), three also underwent cataract surgery; none of the patients administered C_3_F_8_ were involved. The time taken for surgery was about 30 min. The type of illumination included microscope illumination by an OPMI LUMERA 700 (Carl-Zeiss, Germany) and illumination by the 23-G Constellation device (Alcon, Fort Worth, TX, USA). Postoperative treatment consisted of routine topical antibiotics and anti-inflammatory agents for 1 month. The patients were routinely followed up at 1 day, 7 days, 1 month, and 3 months postoperatively. BCVA, intraocular pressure, OCTA (in the C3F8 group, data on OCTA were recorded after complete disappearance of the gas), and complications were routinely monitored during outpatient visits before and after SO removal.

### Examinations

The diagnosis of retinal detachment was initially confirmed by slit lamp biomicroscopy and indirect ophthalmoscopy. Perioperative data were obtained from the medical records, including age, sex, axial length, and duration of SO tamponade. BCVA (LogMAR) and intraocular pressure (IOP) in every patient were measured daily during hospitalization. The Lens Opacities Classification III System (LOCS III system) was used to specify cataract classification.

### Assessment of visual acuity

The BCVA assessment was performed using an international standard visual acuity chart, and data were transformed into the logarithm of the minimum angle of resolution (LogMAR) units.

### OCTA and analysis

**Fig. 1:**
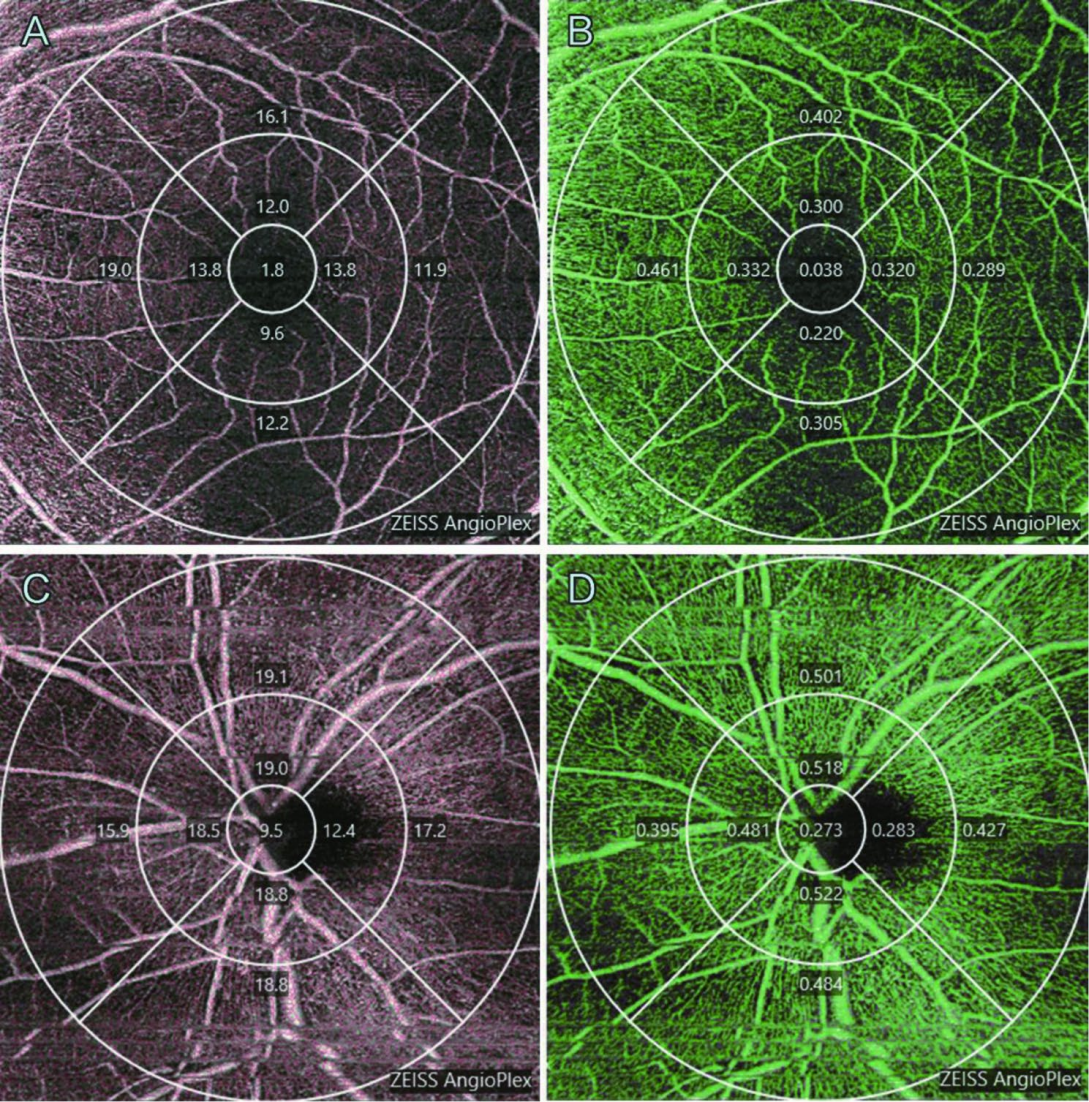
OCT angiography (OCTA) in a 50-year-old male with an eye administered silicone oil (SO) tamponade. The central area (1-mm diameter central circle), the mean and sector values of the inner ring between the 1-mm and 3-mm diameter circles, the outer ring between the 3-mm and 6-mm diameter circles, and the full area (6-mm diameter outer circles) were assessed. Superficial vessel density (SVD) of the macular and peripapillary regions (A and C) and superficial perfusion density (SPD) of the macular and peripapillary regions (B and D) were automatically determined

In this study, 6 × 6 mm^2^ OCTA (Zeiss HD-OCT 5000 with AngioPlex; Carl Zeiss Meditec, Oberkochen, Germany) scans were used for determining SVD and SPD in the macular and peripapillary regions. All scans were obtained by skilled examiners with at least 5 years of OCTA experience and were proficient in conducting OCTA. The SCP comprises the capillaries from the internal limiting membrane (ILM) to the inner plexiform layer (IPL). Scans showing signal strengths > 6 and without motion artifacts or segmentation errors were evaluated [19]. Scan analysis was performed automatically with Cirrus OCTA (AngioPlex 10.0; Carl Zeiss Meditec). VD (in mm^− 1^) and perfusion density (PD; in % converted to decimals) were assessed as previously reported [20]. PD was defined as the total perfused vasculature per unit area in a measurement region. VD was defined as the total length of perfused vasculature per unit area in a measurement region. SVD and SPD were analyzed in the central region (1-mm diameter circle), inner ring between the 1-mm and 3-mm circles, outer ring between the 3-mm and 6-mm circles, and full area (6-mm diameter circle) (Fig. 1), preoperatively, and at 1 day, 7 days, 1 month, and 3 months after SO removal. OCTA was measured in patients with C_3_F_8_ when the C_3_F_8_ had completely disappeared.

### Statistical analysis

Data are shown as means ± standard deviation (SD). Data analysis was performed With SPSS 23.0 (SPSS, USA). One-way analysis of variance (ANOVA) followed by the Bonferroni post hoc test was performed to analyze the differences among multiple independent samples. SVD, SPD and BCVA before and 1 day, 7 days, 1 month, and 3 months after SO removal were compared using repeated measures analysis of variance. Correlations between BCVA and SVD or SPD were assessed using Pearson’s correlation test. *P* < 0.05 indicated statistical significance.

## Results

### Characteristics of the patients

Ninety-four patients were enrolled initially, among whom 54 SOT contralateral eyes with BCVA (LogMAR) < 0.1 and OCTA signal strength > 8 were selected as control eyes. Among the treated eyes, 44 were excluded due to loss to follow-up (n = 21), scans with signal strength < 6 in OCTA (n = 13), ERM (n = 3), macular edema (n = 4), macular hole (n = 1), vitreous hemorrhage (n = 1), and retina redetachment (n = 1). Therefore, 50 eyes from 50 patients (27 males and 23 females; 53.6 ± 10.9 years of age) successfully treated by SO removal between June 2019 and December 2019 were assessed. The participants’ demographic features and preoperative information are shown in Table 1. The mean duration of SO tamponade was 72.4 days. The axial length range was 22–26 mm (mean, 24.33 mm).

**Table 1:** Demographic and presurgical features of the patients with SOT

	SOT eyes	SOT contralateral eyes	PPV + C_3_F_8_ eyes	PPV + C_3_F_8_ contralateral eyes	*P*
Eyes, n	50	54	29	27	
Age, years	53.6 ± 10.9	52.2 ± 11.9	52.6 ± 9.5	53.3 ± 9.4	0. 918
Sex (male/female), n	27/23	34/20	17/12	15/12	0.815
Axial length (mm)	24.24 ± 0.74	24.24 ± 1.42	24.47 ± 0.73	24.54 ± 0.76	0. 493
BCVA, LogMAR BCVA (Snellen)	0.69 ± 0.29 20/79 ± 20/110	0.05 ± 0.067 20/22 ± 20/150	0.434 ± 0.347 20/44 ± 20/80	0.026 ± 0.045 20/21 ± 20/250	< 0.001 < 0.001
Duration of tamponade (months)	3.4 ± 0.6	-	1.6 ± 1.1	-	< 0.001

Data are shown as means ± SD unless otherwise stated. Pre-op, preoperative; BCVA, best-corrected visual acuity; logMAR, the logarithm of the minimum angle of resolution; SOT, silicone oil tamponade; IOP, intraocular pressure; SOR, silicone oil removal

### OCTA parameters of the macular region before SO removal

Compared with the SOT contralateral eyes, eyes with SO tamponade showed reduced SVD and SPD in the macular region (all *P* < 0.01, Supplementary Table S1). The SVD values in the central area, inner ring, outer ring, and full area of the macular region were markedly reduced in the eyes with SO tamponade compared with SOT contralateral eyes (all *P* < 0.01). The SPD values in the central area, inner ring, outer ring, and full area of the macular area also showed marked reductions in eyes with SO tamponade compared with the SOT contralateral eyes (all *P* < 0.01). In addition, SVD values in the central area, inner ring, outer ring, and full area of the macular area from patients with PPV + C_3_F_8_ were 7.075 ± 3.761, 15.662 ± 3.968, 16.397 ± 3.200 and 15.972 ± 3.316, respectively, which were higher than the values obtained for the eyes with SO tamponade (all *P* < 0.05). The SPD values in the central area, inner ring, outer ring, and full area of the macular area of the PPV + C_3_F_8_ eyes were 0.153 ± 0.085, 0.374 ± 0.102, 0.402 ± 0.086 and 0.389 ± 0.087, respectively, which were higher than the values obtained for the eyes with SO tamponade (all *P* < 0.05). No significant differences in SVD and SPD were detected between the PPV + C_3_F_8_ and SOT contralateral eyes.

### OCTA parameters of the peripapillary region before SO removal

The SVD and SPD in the central area of the peripapillary region in the eyes with SO tamponade and SOT contralateral eyes were not significantly different (*P* > 0.05). The SVD values in the inner ring, outer ring, and full area of the peripapillary region in the eyes with SO tamponade showed significant decreases compared with the SOT contralateral eyes (*P* < 0.001) (Supplementary Table S1). In addition, the SPD values in the inner ring, outer ring, and full area of the peripapillary region were lower in the eyes with SO tamponade compared with the SOT contralateral eyes (*P* < 0.01). The SVD values were higher in the inner ring, outer ring, and full area of the peripapillary region of PPV + C_3_F_8_ eyes compared with the eyes with SO tamponade (all *P* < 0.05). The SPD values in the central area, inner ring, outer ring, and full area of the peripapillary region of PPV + C_3_F_8_ eyes were higher than in the eyes with SO tamponade (all *P* < 0.05). There were no significant differences in SVD and SPD between the PPV + C_3_F_8_ and contralateral eyes (all *P* > 0.05).

### OCTA parameters of the macular region of eyes administered SO tamponade and SO removal

**Fig. 2:**
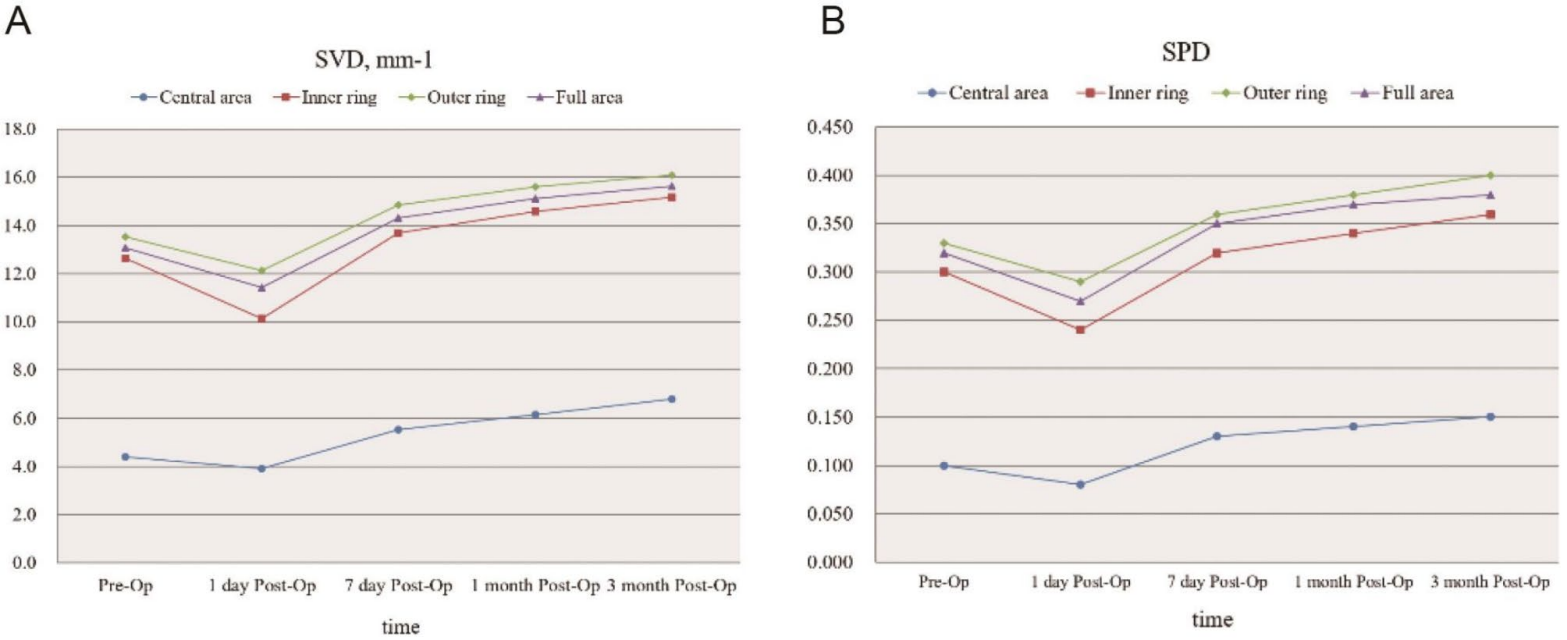
Line charts illustrating SVD and SPD in the macular region before and after SO removal. (A) SVD in the central area, inner ring, outer ring and full area of the macular region. (B) SPD in the central area, inner ring, outer ring and full area of the macular region

After SO removal, both SVD and SPD in the central area, inner ring, outer ring, and full area of the macular region showed significant improvements compared with the preoperative values (repeated measures analysis of variance, *P* < 0.01) (Supplementary Table S1). The pairwise comparisons indicated that SVD and SPD in the inner ring, outer ring, and full area began to increase (all *P* < 0.05) at 1-month post-SO removal, and the central area showed markedly increased values (*P* = 0.04 and *P* = 0.03, respectively) until 3-month post-SO removal. In addition, the SVD and SPD showed trends of time-dependent increase in the macular region from 1 day to 3 months postoperatively (all *P* < 0.001) (Fig. 2). Compared with preoperative values, SVD and SPD showed significant increases at 3 months post-operation (*P* < 0.001). We also found that SVD and SPD in the macular region had no statistically significant differences among 7 days, 1 month, and 3 months (all *P* > 0.05).

### OCTA parameters of the peripapillary region of eyes administered SO tamponade and SO removal

There were significant differences in SVD and SPD in the peripapillary region among the groups (pre-operation and postoperative 1 day, 7 days, 1 month, and 3 months), as shown in Supplementary Table S1 (all *P* < 0.05), but there were no significant differences in SVD and SPD in the peripapillary region between pre-operation and 3 postoperative months (all *P* > 0.05).

### Visual acuity of eyes administered SO tamponade

**Fig. 3:**
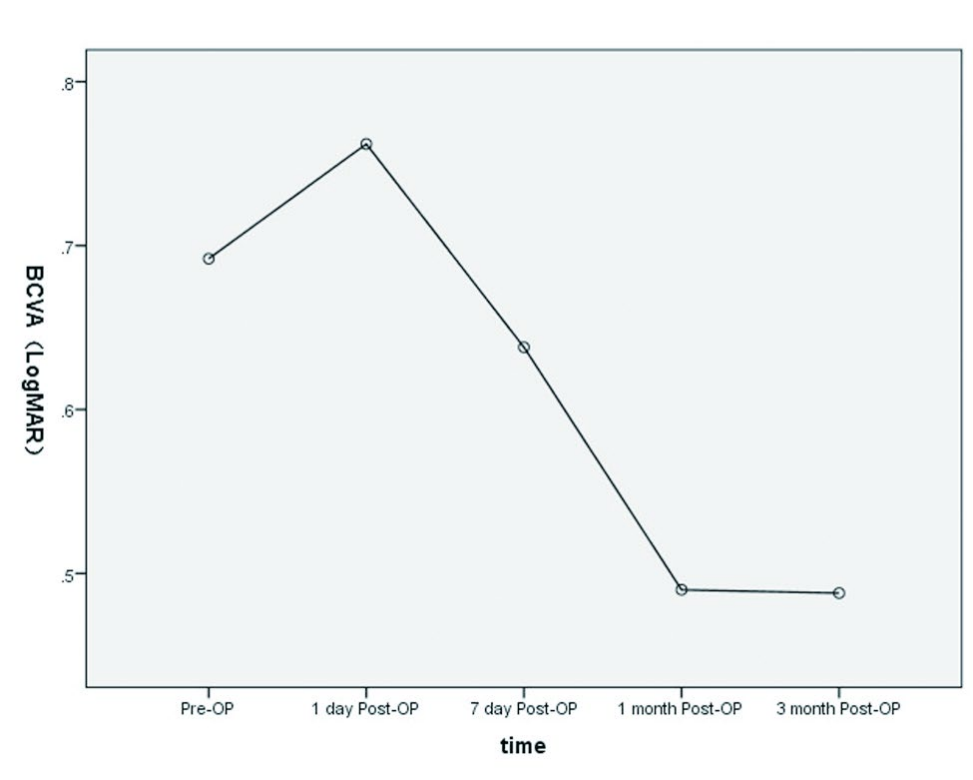
Line chart illustrating BCVA (LogMAR) before and after SO removal. BCVA (LogMAR) showed a trend of time-dependent decrease from postoperative 1 day to 3 months (*P* < 0.001 vs. 1 postoperative day)

The average BCVA (LogMAR) was higher during SO tamponade (0.69 ± 0.27) compared with the SOT contralateral control eyes (0.05 ± 0.07, *P* < 0.001). BCVA (LogMAR) was significantly decreased after SO removal to 0.49 ± 0.32 (*P* < 0.001). In addition, BCVA (LogMAR) showed a trend of time-dependent decrease from postoperative 1 day to 3 months (*P* < 0.001) (Fig. 3). In order to exclude the possible interference of cataract, we performed a separate analysis of BCVA (LogMAR) for the three patients with cataract and the remaining 47 patients before and after SO removal. The results showed significant BCVA (LogMAR) differences in the three patients with cataract surgery from 1 day to 3 months postoperatively (*P* = 0.001); however, there were no significant differences in pairwise comparisons. In the remaining 47 patients, BCVA (LogMAR) values were significantly decreased at postoperative 1–3 months compared with the preoperative values (both *P* < 0.001) (Supplementary Table S2).

### Pearson correlation analysis of BCVA (LogMAR), SVD, and SPD

**Fig. 4:**
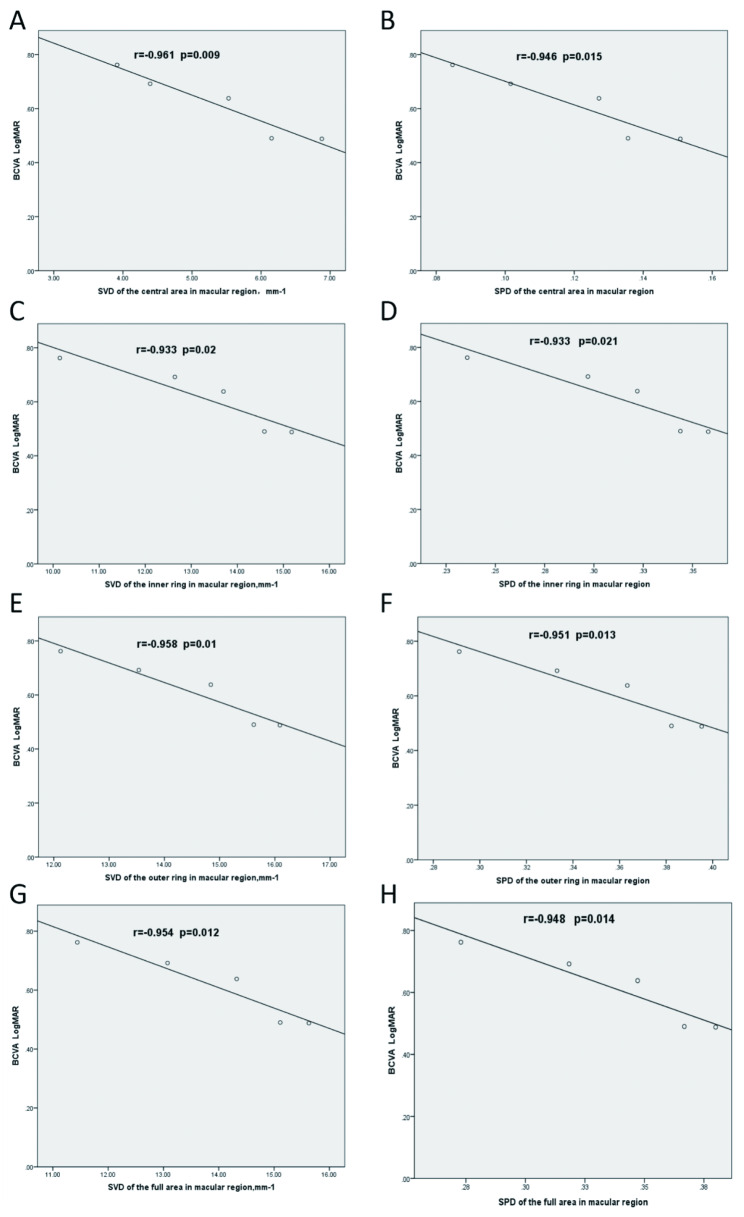
Correlation analysis of BCVA (LogMAR), SVD and SPD in the central area, inner ring, outer ring, and full area of the macular region from pre-operation to 3 postoperative months. (A) BCVA and SVD in the central area of the macular region. (B) BCVA and SPD in the central area. (C) BCVA and SVD in the inner ring. (D) BCVA and SPD in the inner ring. (E) BCVA and SVD in the outer ring. (F) BCVA and SPD in the outer ring. (G) BCVA and SVD in the full area. (H) BCVA and SPD in the full area

In the eyes with SO removal, BCVA (LogMAR) was significantly correlated with SVD and SPD in the central area, inner ring, outer ring, and full area of the macular region. The Pearson correlation test showed that SVD and SPD in the central area, inner ring, outer ring, and full area of the macular region were negatively correlated with BCVA (LogMAR) from pre-operation to 3 postoperative months (all *r*<-0.9, *P* < 0.05; Fig. 4).

## Discussion

The SVD and SPD in the central area, inner ring, outer ring, and full area were lower in the eyes with SO tamponade compared with the control eyes. It is unclear whether the decrease in macular microcirculation was due to RRD damage or SO tamponade duration. Agarwal et al. [21] showed that RRD eyes had decreased microvascular density after successful vitrectomy compared with age-matched healthy cases. Bonfiglio et al. [22] also pointed out that RRD eyes following a successful vitrectomy show reduced vessel density in the SCP and DCP compared with healthy eyes. In contrast, Woo et al. [23] and Yoshikawa et al. [24] held the opposite view. In their studies, postoperative mean SVD values showed no significant differences in RRD patients compared with healthy controls. The reasons for this discrepancy remain unknown, indicating that further investigation is required. In this study, we assessed blood flow changes at 1 day, 7 days, 1 month, and 3 months after the operation, and the 1-month time point seemed optimal for routine examination. At 1 month after SO removal, the SVD and SPD in the macular region were significantly increased compared with the SO tamponade condition. Nevertheless, SVD and SPD in the peripapillary region had no significant increases compared with the preoperative values. Therefore, we might speculate that SO tamponade and SO removal might influence macular microvasculature rather than the peripapillary region.

The present study showed that SO might have a negative long-term effect on retinal microcirculation, as suggested by Kubicka-Trzaska et al. [25]. Nevertheless, the present results contradict previous studies. Indeed, Xiang et al. [17] showed that SO use for < 6 months has no significant effect on macular capillary VD of the SCP and DCP. Meanwhile, Lee et al. [18] showed that SO tamponade duration significantly correlates with VD in the DCP. The possible reasons for the discrepancies between our findings and others’ may include differences in the mean durations of SO tamponade [4.5 ± 1.2 months, 5.6 ± 2.2 months, and 3.4 ± 0.6 months in Lee et al. [18], Xiang et al. [17], and the present study, respectively], the inclusion and exclusion criteria, OCTA devices used, sample size, follow-up period, and study design (retrospective and prospective). Furthermore, SO tamponade may impact the structures and functions of the retina and choroid [13, 26]. In addition, Kubicka-Trzaska et al. [25] found that SO tamponade might influence retinal blood flow and have a negative long-term effect on retinal microcirculation. Therefore, we suggest that SO tamponade affects the macular and peripapillary capillary VDs of the SCP and DCP. Importantly, the present study suggests that SO removal could revert, at least in part, those detrimental changes, but whether those improvements are due to SO itself or other surgical or drug factors remains to be examined.

Our results also showed no significant differences in SVD and SPD of the macular and peripapillary regions between the SOT contralateral and PPV + C_3_F_8_ eyes. PPV + C_3_F_8_ eyes showed significantly higher SVD and SPD in the macular and peripapillary regions than in eyes with SO tamponade. Thus, we infer that the retinal detachment itself and gas tamponade may not affect the early vascular changes significantly, and the effect of SO on the early vascular changes may be specific. It was noted that the duration of C_3_F_8_ tamponade (from operation to examination date) was significantly shorter than in the eyes administered SO tamponade, which might interfere with the results. Whether retinal microcirculation was better in 3 and 6 months after gas tamponade compared with 2 months after gas tamponade needs to be further investigated.

It should be noted that the exact mechanism underlying the increased macular capillary VDs observed after SO removal was not determined. OCTA utilizes optical measurements, and the waterproofing effect of SO seemed to have little impact on the current data. Still, it is worth further discussing this issue with expert physicists. SO filling and removal could affect OCTA data in multiple ways. Firstly, SO exerts pressure on the retina in the sitting, lateral, and prone positions. The pressure of SO on the fovea in the prone position might damage retinal structures and cause ischemia and neuronal damage [27], altering OCTA measurements. The mechanical pressure on the retina is alleviated upon SO removal, helping assess SVD and SPD. Secondly, eyes with SO are very vulnerable to a transient increase in light exposure, which might cause retinal thinning and vascular insufficiency [28, 29]. We infer that, upon SO removal, the threshold for phototoxicity might be restored. Thirdly, the long-lasting K + accumulation in the outer retina and fibrogenic (bFGF) and inflammatory (IL-6) factor accumulation in the retro-SO [30, 31] could damage retinal ganglion cells or foveal microvascular structures during the tamponade. However, further investigation is required, and whether other fluids could have similar effects is unknown.

The present study had limitations. First, DCP and choriocapillaris were not assessed in the macular and peripapillary regions. Further investigation based on swept-source- and projection-resolved OCTA is required to confirm the current findings. In addition, other diagnostic parameters, including retinal thickness, foveal avascular zone (FAZ) area, contrast sensitivity, retinal sensitivity, and ETDRS letter scores, could be included in future studies to analyze the associations among various parameters. Furthermore, the present OCTA study only analyzed 6 × 6-mm^2^ scans; a wider field (e.g., 12 × 12-mm^2^) fovea-centered scan would provide more information about SVD and SPD in patients with SO tamponade. Lastly, we focused on the superficial vessel structure, and the deep plexus was not evaluated.

## Conclusion

This research indicated that SVD and SPD were lower in eyes with SO tamponade than in contralateral eyes. SVD and SPD were increased in the macular region of the eyes with SO tamponade and removal. BCVA (LogMAR) decreased while SVD and SPD increased in the macular region after SO removal; BCVA (LogMAR) was negatively correlated with macular SVD and SPD values, suggesting an improvement in visual acuity after SO removal. Gas tamponade may not significantly affect early vascular changes, but more data are needed to support this notion. Future studies are warranted to explore the mechanism underlying retina microcirculation changes in eyes administered SO tamponade.

## Electronic supplementary material

Below is the link to the electronic supplementary material.

Supplementary Material 1

Supplementary Material 2

## Data Availability

All data relevant to the study are included in the article.
