# Supplementary material for: Early vascular changes after silicone oil removal using optical coherence tomography angiography
Source: BMC Ophthalmol. 2023 Mar 29;23:128. doi: 10.1186/s12886-023-02868-7 (PMC10053480; doi:10.1186/s12886-023-02868-7)
Supplement: Supplementary file 1 — Supplementary Material 1 [file 12886_2023_2868_MOESM1_ESM.docx]

**Supplementary Table S1 SVD and SPD in the macular region and peripapillary region of SOT eyes and PPV+C3F8 eyes**

| **Region** | **PPV+C_3_F_8_**  **Contralateral eyes (n=27)** | **PPV+C_3_F_8_ eyes (n=29)** | **SOT Contralateral eyes**  **(n=54)** | **SOT eyes**  **(n=50, Pre-Op)** | **1 day** | **7 days** | **1 month** | **3 months** |
| --- | --- | --- | --- | --- | --- | --- | --- | --- |
| **Macular region** |  |  |  |  |  |  |  |  |
| SVD, mm^-1^ |  |  |  |  |  |  |  |  |
| Central area | 8.115±2.476 | 7.076±3.761^**^ | 7.928±3.189^##^ | 4.394±3.294 | 3.91±3.13 | 5.53±2.75 | 6.15±3.68 | 6.8±2.95 |
| Inner ring | 17.104±2.398 | 15.662±3.968^**^ | 17.322±2.147^##^ | 12.642±4.642 | 10.15±4.51 | 13.7±3.01 | 14.59±3.7 | 15.18±3.57 |
| Outer ring | 17.538±2.148 | 16.397±3.200^**^ | 17.496±1.721^##^ | 13.538±3.870 | 12.12±3.42 | 14.84±2.65 | 15.62±2.67 | 16.1±2.83 |
| Full area | 17.162±2.145 | 15.972±3.317^**^ | 17.193±1.758^##^ | 13.074±3.907 | 11.44±3.60 | 14.32±2.64 | 15.11±3.05 | 15.63±2.93 |
| SPD |  |  |  |  |  |  |  |  |
| Central area | 0.179±0.058 | 0.153±0.085^*^ | 0.174±0.077^##^ | 0.102±0.093 | 0.08±0.07 | 0.13±0.07 | 0.14±0.09 | 0.15±0.07 |
| Inner ring | 0.407±0.061 | 0.374±0.102^**^ | 0.412±0.053^##^ | 0.297±0.115 | 0.24±0.11 | 0.32±0.07 | 0.34±0.09 | 0.36±0.09 |
| Outer ring | 0.432±0.055 | 0.402±0.086^**^ | 0.431±0.045^##^ | 0.333±0.101 | 0.29±0.09 | 0.36±0.07 | 0.38±0.08 | 0.40±0.07 |
| Full area | 0.420±0.054 | 0.389±0.087^**^ | 0.420±0.045^##^ | 0.318±0.101 | 0.27±0.09 | 0.35±0.07 | 0.37±0.08 | 0.38±0.07 |
| **Peripapillary region** |  |  |  |  |  |  |  |  |
| SVD, mm-1 |  |  |  |  |  |  |  |  |
| Central area | 5.083±3.781 | 5.304±3.979 | 4.302±2.702 | 3.430±3.351 | 3.83±2.21 | 5.04±3.59 | 5.28±3.81 | 4.77±2.75 |
| Inner ring | 17.417±2.039 | 16.686±2.750^**^ | 17.600±1.728^##^ | 14.066±4.457 | 13.65±3.33 | 15.83±2.16 | 15.76±3.28 | 15.28±3.35 |
| Outer ring | 18.270±1.769 | 16.707±2.968^**^ | 17.983±1.923^##^ | 14.284±4.357 | 12.62±3.79 | 15.71±2.28 | 15.94±3.05 | 15.9±2.79 |
| Full area | 17.700±1.566 | 16.375±2.616^**^ | 17.506±1.604^##^ | 13.910±4.123 | 12.6±3.48 | 15.44±1.94 | 15.6±2.92 | 15.45±2.7 |
| SPD |  |  |  |  |  |  |  |  |
| Central area | 0.140±0.107 | 0.147±0.111 | 0.116±0.075 | 0.091±0.095 | 0.1±0.06 | 0.14±0.1 | 0.15±0.11 | 0.13±0.08 |
| Inner ring | 0.454±0.056 | 0.434±0.075^**^ | 0.460±0.046^##^ | 0.366±0.123 | 0.35±0.82 | 0.41±0.06 | 0.41±0.09 | 0.40±0.09 |
| Outer ring | 0.458±0.047 | 0.417±0.082^*^ | 0.450±0.051^##^ | 0.357±0.116 | 0.32±0.1 | 0.39±0.06 | 0.4±0.08 | 0.4±0.07 |
| Full area | 0.448±0.042 | 0.415±0.072^**^ | 0.443±0.043^##^ | 0.352±0.111 | 0.32±0.09 | 0.39±0.05 | 0.4±0.08 | 0.39±0.07 |

Values are shown as mean ± SD. SVD, superﬁcial vessel density; SPD, superﬁcial perfusion density; SOT, silicone oil tamponade; PPV+C3F8: pars plana vitrectomy and perfluoropropane; OCTA, optical coherence tomography angiography. 1 day: day 1 after SOR; 7 days: day 7 after SOR; 1 month: one month after SOR; 3 months: 3 months after SOR.

^#^*P*<0.05 and ^##^*P*<0.01 Control eye vs. SOT eye.

^*^*P*<0.05 and ^**^*P*<0.01 PPV+C3F8 eye vs. SOT eye.

^$^*P*<0.05 and ^$$^*P*<0.01 Contralateral eye vs. PPV+C3F8 eye.

^&^*P*<0.05 and ^&&^*P*<0.01 Control eye vs. Contralateral eye.
