# Supplementary material for: Early vascular changes after silicone oil removal using optical coherence tomography angiography
Source: BMC Ophthalmol. 2023 Mar 29;23:128. doi: 10.1186/s12886-023-02868-7 (PMC10053480; doi:10.1186/s12886-023-02868-7)
Supplement: Supplementary file 2 — Supplementary Material 2 [file 12886_2023_2868_MOESM2_ESM.docx]

**Supplementary Table S2 BCVA (LogMAR) values before and after SOR in patients with or without cataract surgery**

|  | | **BCVA (logMAR), mean ± SD** | | | | | |
| --- | --- | --- | --- | --- | --- | --- | --- |
|  |  | **Pre-Op** | **1 day** | **7 days** | **1 month** | **3 months** | ***P*** |
| Patients with cataract (n=3) | BCVA (logMAR and Snellen) | 0.77±0.21  (20/109±20/262) | 0.93±0.12  (20/171±20/693) | 0.73±0.06  (20/109±20/693) | 0.53±0.15  (20/67±20/200) | 0.53±0.15  (20/67±20/200) | 0.002 |
| Patients without cataract (n=47) | BCVA  (logMAR and Snellen) | 0.68±0.3  (20/78±20/108) | 0.75±0.3  (20/89±20/119) | 0.63±0.29#  (20/70±20/101) | 0.48±0.32*#^  (20/47±20/71) | 0.48±0.32*#^  (20/47±20/68) | <0.001 |

**P*<0.001 vs. Pre-Op; #*P*<0.001 vs. day 1 after SOR. ^*P*<0.001 vs. day 7 after SOR.
